# Refractory hyponatremia secondary to idiopathic, isolated aldosterone deficiency

**DOI:** 10.1093/omcr/omag062

**Published:** 2026-05-10

**Authors:** Noah W Free, Anastasia E Metropulos, Jeremy R Williams, Prakash Balasubramanian, Daniel R Principe

**Affiliations:** Department of Medicine, University of Wisconsin, Madison, WI 53792, USA; Indiana University School of Medicine, Gary, IN 46408, USA; Department of Medicine, University of Wisconsin, Madison, WI 53792, USA; William S. Middleton Memorial Veterans Hospital, Madison, WI 53792, USA; Department of Medicine, University of Wisconsin, Madison, WI 53792, USA

**Keywords:** refractory hyponatremia, aldosterone, endocrinology

## Abstract

Hypoaldosteronism is an uncommon, but well-recognized cause of hyponatremia. Hypoaldosteronism is often attributed to renin deficiency or primary adrenal insufficiency. Here, we present the case of a patient with recurrent, refractory hyponatremia due to hypoaldosteronism with normal serum renin activity and cortisol levels. He was started on fludrocortisone with normalization of electrolytes. This is a highly unusual case of hypoaldosteronism without perturbation to the renin-angiotensin system or adrenal insufficiency and serves as an important reminder that aldosterone deficiency should be considered as a potential cause of hyponatremia independent of renin activity or other markers of adrenal function.

## Introduction

Hyponatremia is the most common electrolyte abnormality in hospitalized patients. Mild hyponatremia can present with vague, non-specific symptoms including myalgias, nausea/vomiting, and headache [1]. If untreated, severe hyponatremia can cause life-threatening complications, notably rhabdomyolysis, altered mental status, seizure, coma, and death [1]. Common causes of hyponatremia include medications, syndrome of inappropriate antidiuretic hormone release (SIADH), recurrent vomiting and/or diarrhea, heart failure, and renal/liver disease [1]. Hypoaldosteronism is also a well-recognized cause of hyponatremia. Aldosterone is a mineralocorticoid hormone produced in the adrenal cortex that regulates total body water and sodium balance by increasing both sodium and water reabsorption as well as excretion of potassium [2]. In adults, new onset hypoaldosteronism occurs primarily in the setting of either renin deficiency [3] or primary adrenal insufficiency [4]. Here, we present the unusual case of a patient presenting with recurrent, refractory hyponatremia attributed to isolated hypoaldosteronism with no additional abnormalities to the renin-angiotensin system and no other laboratory findings suggestive of adrenal insufficiency.

## Case report

A 71-year-old man presented to the emergency department (ED) with malaise, nausea, and vomiting for the past day. His medical history was significant for hypertension, hyperlipidemia, microscopic colitis, and takotsubo cardiomyopathy now resolved. He had an active diagnosis of non-small cell lung cancer status post upper lobe wedge resection two years ago, having completed four cycles of carboplatin/gemcitabine and adjuvant pembrolizumab for one year that finished two months prior to presentation. He was receiving radiation but no other systemic cancer therapy. Of note, he had multiple admissions for hyponatremia in the last five months, each in the setting of diarrhea and dehydration. His serum sodium ranged from 116–130 mEq/l, though serum and urine osmolality and urine sodium were highly variable. As he was not taking any medications known to cause hyponatremia, the working theory was that his hyponatremia was due to a combination of malignancy associated SIADH superimposed with hypovolemic hyponatremia from gastrointestinal losses. Each admission, his sodium corrected with normal saline infusion, though at his most recent hospitalization, he was started on salt tabs for persistent hyponatremia despite volume resuscitation. The day of the present admission, he felt generally unwell and endorsed several episodes of vomiting and came ED for further evaluation.

On arrival to the ED, vitals were stably normal. He was clinically hypovolemic dry mucous membranes, but his exam was otherwise unremarkable. Laboratory studies demonstrated hyponatremia of 122 and mild hyperkalemia (Table 1). He was given multiple normal saline boluses with improvement in his sodium to 125. The next morning, his serum sodium decreased to 123, and urine sodium was elevated to 116. Presuming a diagnosis of SIADH, he was placed on a 1.5 liter fluid restriction and salt tabs were increased to 6 g three times a day, though he was unable to tolerate this dose and was transitioned to 15 mg of oral urea daily. Despite an initial improvement, his serum sodium rapidly down trended to a nadir of 116 requiring hypertonic saline infusion.

**Table 1:** Pertinent lab values from our patient’s initial workup.

Laboratory Study	Value	Reference Range
Sodium	122 mEq/l	135–145 mEq/l
Potassium	5.3 mEq/l	3.5–5.1 mEq/l
Chloride	90 mEq/l	98–107 mEq/l
CO₂	26 mEq/l	22–29 mEq/l
Blood Glucose	110 mg/dl	70–99 mg/dl
BUN	22 mg/dl	7–20 mg/dl
Creatinine	1.62 mg/dl	0.6–1.3 mg/dl
Aldosterone	<1 ng/dl	3–16 ng/dl
Renin Activity	3.45 ng/ml/hr	0.6–4.3 ng/ml/hr
Renin:Aldosterone Ratio	Incalculable	<20–30
Morning Cortisol	14.88 μg/dl	5–25 μg/dl
Adrenocorticotropic hormone	19 pg/ml	7–63 pg/ml
Thyroid Stimulating Hormone	2.694 μIU/ml	0.4–4.5 μIU/ml
Anti-21-hydroxylase antibody	<0.1 U/ml	<0.1 U/ml
Urine Sodium	136 mEq/l	20–110 mEq/l
Urine Potassium	33 mEq/l	12–62 mEq/l
Urine Sodium:Potassium Ratio	4.12	1–3
Urine Osmolality	743 mOsm/kg	300–900 mOsm/kg

Table showing the patient’s initial metabolic labs and urine studies, as well as subsequent workup used to diagnose an isolated aldosterone deficiency.

**Figure 1:**
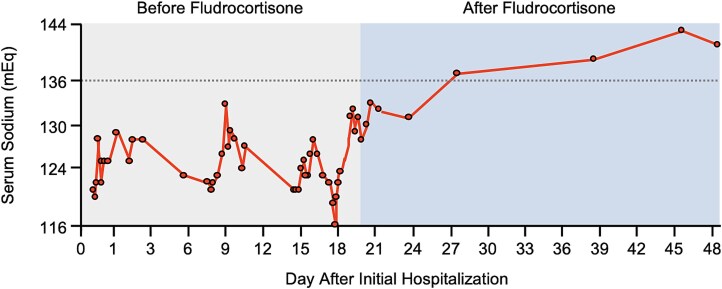
Serum sodium values over time. Sodium values are shown starting at the day of our patient’s initial presentation. The dotted line shows the initiation of fludrocortisone 0.1 mg, which was uptitrated to 0.2 mg after three days.

As his electrolyte derangements persisted despite clinical euvolemia, improved oral intake, and was refractory to aggressive therapies directed toward SIADH, we considered additional etiologies. His persistent hyperkalemia despite improvement in renal function and volume status raised concern for hypoaldosteronism, further supported by marked elevation of his urine sodium to potassium ratio. Given his prior exposure to immune checkpoint inhibitors, we first performed comprehensive testing to rule out primary adrenal insufficiency. However, laboratory testing showed normal morning cortisol and negative anti-21-hydroxylase antibody suggesting against this etiology. Importantly, his workup was also notable for high-normal renin activity and undetectable serum aldosterone levels despite repeat testing more suggestive of an isolated aldosterone deficiency (Table 1). Hence, he was trialed on 0.1 mg fludrocortisone daily, with stabilization of serum sodium in the mid-120s. Fludrocortisone was increased to 0.2 mg daily after three days, after which his sodium values improved and remained stable from 130–137. He was discharged in stable condition with close nephrology follow up. At subsequent visits, our patient stated that he felt much better and that his symptoms had resolved completely. In the 18 months since discharge, his electrolytes have all remained within normal limits (Figure 1) and he has not required additional hospitalization.

## Discussion

Here, we present the case of a patient with recurrent, refractory hyponatremia due to an isolated and idiopathic aldosterone deficiency. As discussed, hypoaldosteronism is a well-recognized cause of hyponatremia, commonly due to either renin deficiency or primary adrenal insufficiency [3, 4]. In hyporeninemic hypoaldosteronism, both serum renin activity and serum aldosterone levels would be subnormal and fail to increase with sodium depletion [3]. This is typically observed in the setting of either diabetic nephropathy, calcineurin inhibitors, or frequent NSAID use, none of which pertains to our patient [5]. Hence, given the lack of these conditions and our patient’s normal renin activity assay, his presentation was inconsistent with hyporeninemic hypoaldosteronism. Similarly, given his persistently normal morning cortisol levels and negative anti-21-hydroxylase antibody, his presentation was also inconsistent with primary adrenal insufficiency.

There are also reports of aldosterone deficiency in the setting of critical illness [6], as well as medication-induced suppression of aldosterone synthesis, most notably regarding angiotensin-converting enzyme (ACE) inhibitors [7] or chronic use of heparin products containing chlorbutol [8]. However, none of these apply to our patient and the etiology of his hypoaldosteronism remains unclear. Though it is unlikely we will conclusively identify the cause of his hypoaldosteronism, there are isolated observations that may provide some potential insights into our patient’s presentation. For example, it has been suggested that in cancer patients, interleukin-6 inhibits secretion of the aldosterone by adrenal tissue [9]. Hence, interleukin-6 and other upstream regulators of aldosterone production/secretion warrant additional investigation.

In summary, here we present the case of a patient with an isolated and undifferentiated aldosterone deficiency leading to refractory hyponatremia. Though less common, hypoaldosteronism should be considered in patients with hyponatremia, particularly those with increased urine sodium and osmolality in the absence of other etiologies including salt wasting nephropathies or cerebral salt wasting. Additional clues suggestive of hypoaldosteronism are hyperkalemia, hyperchloremic non-anion gap metabolic acidosis, and low fractional excretion of potassium as evidenced by a high urine sodium to potassium ratio [10]. This case underscores our limited understanding of aldosterone regulation and serves as an important reminder that aldosterone deficiency should be considered as a potential cause of hyponatremia independent of other markers of adrenal function.
